# Supplementary material for: Analysis of Single- and Double-Stranded DNA Damage in Osteoblastic Cells after Hyperbaric Oxygen Exposure
Source: Antioxidants (Basel). 2023 Apr 1;12(4):851. doi: 10.3390/antiox12040851 (PMC10135236; doi:10.3390/antiox12040851)
Supplement: Supplementary file 1 [file antioxidants-12-00851-s001.zip › antioxidants-2280484-supplementary.pdf]

Supplemental Table S1. List of primers and annealing temperatures used for qRT-PCR.

Gene of interest	primer (5′ » 3′)	annealing temp./time	company
GAPDH	Fw: TCCATGACAACTTTGGTATCGTGG	58 °C, 20 sec	Eurofins Genomics
	Rv: GACGCCTGCTTCACCACCTTCT	58 °C, 20 sec	Eurofins Genomics
HO-1	Fw: TCCGATGGGTCCTTACACTC	55 °C, 20 sec	Eurofins Genomics
	Rv: TAAGGAAGCCAGCCAAGAG	55°C, 20 sec	Eurofins Genomics
NQO1	Fw: AAAAGAAGCTGGAAGCCGCA	58 °C, 45 sec	realtimeprimers.com
	Rv: AGGATTTGAATTCGGGCGTC	58 °C, 45 sec	realtimeprimers.com
TGF-ß1	Fw: CGTGGAGCTCTACCAGAAATA	58 °C, 20 sec	Eurofins Genomics
	Rv: TCCGGTGACATCAAAAGATAA	58 °C, 20 sec	Eurofins Genomics
